# Recycled PLA for 3D Printing: A Comparison of Recycled PLA Filaments from Waste of Different Origins after Repeated Cycles of Extrusion

**DOI:** 10.3390/polym15173651

**Published:** 2023-09-04

**Authors:** David Hidalgo-Carvajal, Álvaro Hortal Muñoz, José J. Garrido-González, Ruth Carrasco-Gallego, Victoria Alcázar Montero

**Affiliations:** 1 Escuela Técnica Superior de Ingenieros Industriales, Universidad Politécnica de Madrid, 28006 Madrid, Spainruth.carrasco@upm.es (R.C.-G.); 2 Dirección de Compras Industrial y Cliente, Repsol, 28006 Madrid, Spain; 3 Facultad de C. Químicas, Universidad de Salamanca, 37008 Salamanca, Spain; 4 Grupo de Investigación Polímeros, Caracterización y Aplicaciones (POLCA), 28006 Madrid, Spain

**Keywords:** recycled PLA filaments, re-extrusion cycles, circular economy, distributed recycling, DOSY

## Abstract

The objective of this work is to evaluate the reprocessing of PLA 3D printing waste from different origins, into filaments and films, and without the addition of any additive. Two types of waste were considered: a blend of different printing wastes (masks, visors, other components) of personal protective equipment coming from an association of Spanish coronamakers, and PLA waste from a single known commercial source. Both types of materials were subjected to repeated extrusion cycles and processed into films by compression molding. Samples were characterized after each cycle and their mechanical and viscosity properties evaluated. Diffusion-ordered NMR spectroscopy (DOSY) experiments were also carried out to estimate molecular weights. The results show a better performance for the PLA waste from the known origin, capable of withstanding up to three re-extrusion cycles per two for the waste blending, without significant degradation. Additionally, a model to address collection and mechanical recycling cycles under two different scenarios (full traceability and not full traceability) was proposed.

## 1. Introduction

3D printing is an additive manufacturing (AM) technique that in recent years has become very attractive, as shown by the expanding market size, projected to grow from USD 15.1 billion in 2021, to USD 44.5 billion in 2026, at a compound annual growth rate of 24% [1]. Ease of manufacturing, complex geometry designs, reduced times and on-demand production are some of the reasons for the increasing use of additive manufacturing (AM) [2,3,4,5,6]. Different methods of additive manufacturing (AM) have been developed, including stereolithography [7], selective laser sintering [8], inkjet printing [9], laminate object manufacturing [10] and fused deposition modelling (FDM) or fused filament fabrication (FFF) [11]. The most widely used FDM/FFF additive technology uses a thermoplastic filament which is continuously fed into the printer, heated until it melts and afterwards, extruded through a heating nozzle and deposed on a printing platform. Although 3D printing may be considered a more sustainable manufacturing method due to reduced raw and waste materials, less post processing and potential to recycle, its proliferation has raised several environmental concerns [12], as plastic pollution has continued to grow at a dangerous rate.

According to the latest report by the Organization for Economic Cooperation and Development (OECD) a person generates between 69 kg and 221 kg of plastic waste per year, from which about only 9% is properly recycled, 19% incinerated 49% landfilled and, more worrisome, about 22% is mismanaged and becomes uncollected litter [13]. Interestingly, the report also found that during the COVID-19 pandemic period, plastic use decreased by 2.2% during 2020, but it also created an increase in littering from packaging and personal protective equipment (PPE) related to healthcare. Moreover, when the lockdowns ended and economic activities resumed, plastic pollution surged once again. Since most plastics currently in use originate from crude oil after a primary transformation, being considered as virgin and non-degradable materials, the fact that only a small portion is being recycled calls for urgent action. On the other hand, plastic materials produced from renewable sources, such as biomass [14], natural biopolymers [15], or generated through other processes such as chemical synthesis [16], or by fermentation [17], are also increasing, reaching a share of roughly 2% of the total of plastics produced in the world [18]. Potentially, this growth could be beneficial as it “solves” both the non-renewable and the non-degradable parts of the problem, but it still does not solve the need to maximize the use of the material beyond its initial life cycle. In both cases, plastic pollution related to non-degradable and biodegradable materials urges a change from the linear production model, where the material is discarded as waste after its use and added to the biodisagreeable mountain of plastic pollution, towards a circular (economy) model, where the material is used and reused through several stages and cycles, until it reaches an End of Life (EoL) stage. Although the circular model represents a solution which supports economic growth while decreasing environmental impact [19], it is worth noting that this transition requires changes at all levels along the entire model, from material producers improving the material circularity [20], to consumers preventing and reducing the amount of waste generated [21], as well as investment in infrastructure and technology to improve waste management [22], and changes in regulations and policies [23]. Although gradual improvements in the model can be seen nowadays, the low recycling rates and lack of planning for further reintroduction of the materials into the supply chain prove that there is still a long road to walk.

More recently, important changes in product manufacturing processes and technologies towards more sustainable ones have been observed [24], with special focus on those able to combine reduced waste and the possibility to manufacture customized products closer to the consumer [25], such as additive manufacturing through three-dimensional (3D) printers. Sustainability on 3D-printing can be approached from several angles: manufacturing energy and waste, transportation, recycling, and use of bioplastics as raw materials [26]. Moreover, 3D printing technology has the potential to complement the circular economy model by using filaments created from post-consumer recycled polymers [27] to manufacture new products. On this matter, a large body of research has focused on the recyclability of the material [28,29] and its use as raw material after its typical EoL stage [30], despite the challenges it faces regarding deterioration after mechanical recycling.

**Figure 1:**
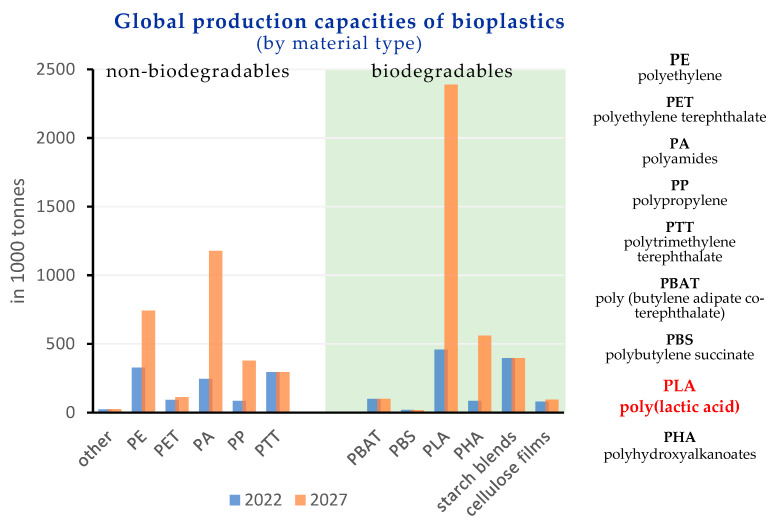
Bioplastic market data (adapted from [43]).

The most used thermoplastics in 3D printing are: poly(lactic acid) (PLA) [31,32,33,34,35], acrylonitrile butadiene styrene (ABS) [36,37], acrylonitrile styrene acrylate (ASA) [38], polyethylene terephthalate (PET) [39], nylon [40], polycarbonate (PC) and thermoplastic polyurethanes (TPU) [41]. Besides, PLA, the so-called “polymer of the 21st century” [42], has become the most widely used biopolymer with a production capacity projected to increase to around 2.4 million tonnes in 2027, as shown in Figure 1 [43]. PLA is biodegradable, biocompatible, recyclable, and compostable; it is commercially available in large scale and can be processed with the usual polymer processing techniques (extrusion, injection molding, etc.). Due to its mechanical, optical, and barrier properties, PLA can be an alternative in many applications to petroleum-based polymers [44].

In addition to its recognized properties (biodegradable, bio-based and biocompatible), PLA is particularly suitable for 3D printing due to its low melting point (150–160 °C), easy processability and acceptable mechanical properties [31], making this polymer one of the preferred materials for FDM/FFF printers in office and home environments. In commercial printers, PLA has found a wide application in the medical and biomedical sectors, due to its biocompatibility, being used in customized anatomical models, surgery equipment, bioprinting, scaffolds for tissue engineering or controlled drug delivery systems [32]. Other uses of PLA in 3D printing are related to the manufacture of agricultural instruments, laboratory equipment and teaching aids [33].

Although 3D printing is considered a sustainable manufacturing method, its expected growth could generate a waste management problem in the future (failed prints, support structures, disposable prototypes, etc.); thus, recycling 3D printing materials is one of the current research trends [44,45,46] to reduce generation of waste and costs (product, feedstock). Thus, in the context of a circular economy, 3D printing with PLA offers an excellent opportunity to comply with the rule of the 3Rs: reduce, reuse, and recycle. To date, some studies have investigated the closed-loop recycling potential of PLA waste generated by FFF printers, that is, the production of new filaments from 3D printing waste. Anderson et al. [47] proposed the direct recycling of PLA waste: the collected wastes were ground and re-extruded into filaments. The average mechanical properties of the recycled PLA specimens after one recycling cycle were lower than those of the virgin specimens (11% for tensile yield strength and 5% for tensile modulus). Cruz-Sanchez et al. [48] evaluated the degradation of the mechanical properties of the PLA after five recycling cycles, considering four different recycling process chains. In another study Zhao et al. [49] found that the PLA can only be reprocessed for two 3D printing cycles due to a rapid decrease in the viscosity values. Although mechanical properties did not significantly change, the reduction in molecular weight during reprocessing cycles was, according to the authors, responsible for the limited reprocessing cycles. Recycling of PLA wastes from university 3D printing laboratories was investigated by Choo et al. [50], suggesting, in this case, that PLA wastes were to be recycled only once.

All these studies have shown the technical feasibility of using recycled PLA for FFF, but also that mechanical recycling of PLA is strongly influenced by the processing parameters and the PLA post-consumer waste. Moreover, despite the potential of closed-loop recycling of PLA 3D printing waste, the use of recycled filaments still presents some limitations regarding the quality of the 3D printed parts [51], even though recycled PLA filaments for 3D printers are commercially available [52].

Moreover, 3D printing is not the only application for recycled 3D printed waste. For instance, assorted leftover PLA from 3D printing has been used to produce composites with a recycled PLA matrix reinforced with carbon fiber [53] or silica particles [54]. Other authors, such as Beltrán et al. [27,55], evaluated the mechanical recycling of PLA wastes from 3D printing to investigate the feasibility to convert those wastes into useful films. A total of two PLA 3D printing wastes were used in the study: wastes from a well-known reference grade and wastes from a mixture of different PLA grades. Films with good performance were obtained when wastes from the well-known PLA grade were used and subjected to a demanding washing stage, a single extrusion cycle, and compression molding [27]. 

The main objective of this work was to compare the mechanical recycling of PLA 3D printing waste from two different origins, for use as filaments in 3D printers and for transformation into films. For this purpose, two different types of waste were used: one came from our university laboratory, so both the commercial filament and the employed 3D printer were known (PLA-C); the other material was obtained from a mix of 3D printing parts generated for personal protective equipment during the COVID-19 pandemic (PLA-M) and neither the filaments nor printers were known. Both wastes were subjected to repeated extrusion cycles, and the effect of reprocessing on the properties of materials was investigated. Fourier-transform infrared (FTIR) spectra were obtained for the structural characterization of the two PLA types (PLA-C and PLA-M) after each reprocessing cycle. Solution viscometry and diffusion-ordered NMR spectroscopy [56] were used to evaluate the effect of mechanical recycling on the molecular weight of the materials. Additionally, after each extrusion cycle, films were prepared by compression molding and their mechanical properties studied. 

FTIR spectra for both PLA types displayed similar transmittance bands, which were characteristic for PLA, and no significant differences along reprocessing were observed. The reduction of molecular weight, after each extrusion cycle, allowed us to conclude that recycling wastes into filaments for 3D printers seems feasible up to three extrusion cycles for PLA-C and two for PLA-M, showing a better performance for the PLA waste from the known origin. 

Finally, a model to review the different collection and mechanical recycling cycles under the two scenarios (full traceability and not full traceability) was proposed. 

## 2. Materials and Methods

### 2.1. Materials

Two different PLA 3D printing wastes have been used. The first material came from discarded parts and 3D printing leftovers from a known commercial PLA [57]. This PLA grade has a density of 1.24 g/cm^3^ and is a black color. The second material was a mixture of different 3D printing wastes, coming from an association of coronamakers (Madrid, Spain) that were used in protective screens during COVID-19. The origin of these printing wastes is unknown, and the color is white, in different shades.

### 2.2. 3D Printing of the Reference Grade

The PLA reference grade was printed using an ABAX PRI5 3D printer, operating at 200 °C, with a bed temperature of 50 °C. 

### 2.3. Processing and Reprocessing of PLA

**Figure 2:**
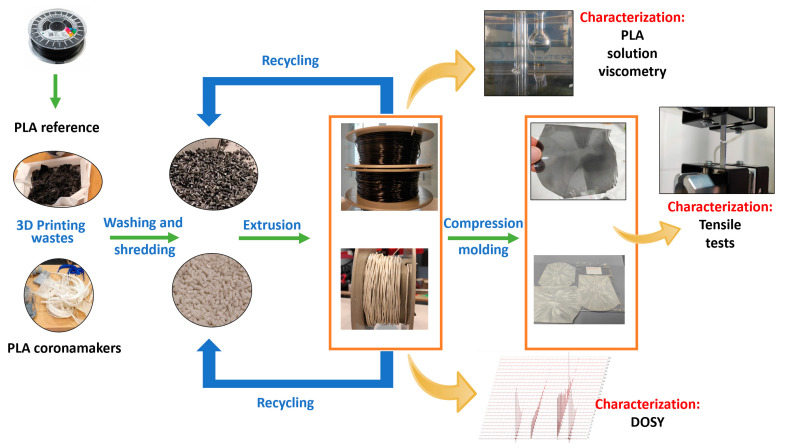
Processing and reprocessing of PLA from 3D printing wastes.

A scheme for the mechanical recycling process is illustrated in Figure 2.

In this experimental work, before processing, both printing wastes were cut into manageable pieces and washed at 60 °C for 15 min in an aqueous solution of the surfactant Triton X-100 (0.3% wt.). The washed materials were dried in a vacuum oven prior to processing. Next, the small pieces were shredded (SHR3D IT shredder, 3devo, Utrecht, The Netherlands) and the granulates dried to remove all traces of moisture; an Airid polymer dryer was used, working at a temperature of 60 °C for 3 h, while the internal screw rotated at 10 revolutions per minute to keep the granulate moving. Last stage of the mechanical recycling was extrusion: PLA filaments were obtained using the Next 1.0 single-spindle extruder (3devo, Netherlands). The temperature profile was 170 °C (hopper), 185 °C, 190 °C and 195 °C (die). One part of extruded filaments was separated for characterization and preparation of films by compression molding and the remaining material was subjected to another processing cycle in identical conditions. 

Films were obtained by compression molding using an IQAP-LAP hot-plate press. The following process conditions were applied, beginning with a melting cycle at 190 °C with no pressure, for 2 min, followed by a degasification step and compression molding at 14 MPa for 4 min. Finally, after a cooling cycle at 14 MPa for 6 min) films were obtained. 

PLA samples have been labelled as follows, **i-PLA-C** and **i-PLA-M**, with 1≤i≤5 representing the number of extrusion cycles during reprocessing; **C** indicates that the 3D printing waste comes from a commercial (known) PLA filament while **M** means that the printing waste comes from a mixture of parts (masks, visors, other components) of the personal protective equipment (PPE) generated by the coronamakers. The samples used in this study are summarized in Table 1. Degradation of the materials did not allow further cycles of extrusion.

### 2.4. Fourier-Transform Infrared Spectroscopy (FTIR)

Fourier-transform infrared spectroscopy (FTIR) spectra were recorded using a Nicolet IR100 (Thermo Fisher Scientific, Madrid, Spain) in a wavenumber range of 600–4000 cm^−1^ in the transmittance mode. Samples were obtained as films by evaporation of solutions in methylene chloride. All the spectra were corrected and normalized at 1454 cm^−1^, assigned to the asymmetric bending of CH_3_ group and known to be a suitable internal standard for PLA [58].

### 2.5. Intrinsic Viscosity of PLA Solutions in Chloroform

The capillary viscosity of PLA samples was measured using an Ubbelohde viscometer in a thermostatic water bath at 25 °C. For each sample, four different concentrations were used, in the range of 0.006 to 0.003 g/mL. Plots of the reduced viscosity ($\eta_{red})$ and the inherent viscosity ($\eta_{inh})$ versus concentration (c) allowed us to obtain the intrinsic viscosity ($\left[\eta \right])$ by extrapolation to zero concentration (intercept with the y-axis). The Mark–Houwink–Sakurada Equation (1) was used to determine the viscosity-average molecular weight ($\bar{M_{\eta }})$ of polymers through the measurement of intrinsic viscosity:

(1) $$\left[\eta \right]=K\times {\bar{M_{\eta }}}^{a}\;\left(dL/g\right)$$

Although literature reports numerous Mark–Houwink constants for poly(lactic acid) in different solvents and temperatures [59], in this study the following values $K=2.21\times 10^{-4}$ and a=0.77 have been used, according to the reported values for dilute PLA solutions in chloroform at 25 °C [60,61,62].

### 2.6. Diffusion-Ordered (DOSY) Nuclear Magnetic Resonance (NMR) Spectroscopy 

Diffusion-Ordered Spectroscopy (DOSY) determines the diffusion coefficients of a species with high accuracy, from the individual resonances in the ^1^H NMR spectrum [63,64]. The diffusion coefficient allows one to estimate the polymer hydrodynamic radius ($r_{h})$ through the Stokes−Einstein Equation (2)

(2) $$D=\frac{k_{B}T}{6\pi \eta r_{h}}$$

where $k_{B}$ is Boltzmann constant, *T* is temperature and η the bulk viscosity of the solvent. The polymer hydrodynamic radius ($r_{h})$ can be correlated to the average molecular weight of the polymer $\left(M\right)$ through the empirical Rouse–Zimm model (3)

(3) $$r_{h}\sim bM^{\alpha }$$

in which b and α are arbitrary parameters, combination of Equations (2) and (3) yields Equation (4) where the diffusion coefficient $\left(D\right)$ and the molecular weight $\left(M\right)$ are correlated [65]:

(4) $$D=AM^{-\alpha }$$

with *A* as an adjusted proportionality factor. Taking logarithm of both sides, Equation (4) is linearized to produce Equation (5):

(5) logD=logA−αlogM

The diffusion coefficients $\left(D\right)$ can be measured via NMR, using a specialized DOSY experiment, the Pulsed Gradient Spin Echo (PGSE) experiment. Several groups have shown that DOSY can be indeed be used to determine the average molecular weights of polymers [66,67]. Nonetheless, despite the advantages of DOSY experiments over size exclusion chromatography (SEC) for molecular weight characterization, such as low sample concentration, minimum amount of solvent, rapid and simple procedure, DOSY has been applied so far to a limited number of polymers [68]. Trying to broaden the spectrum of polymers in which DOSY has been applied, in this study we have used diffusion-ordered NMR spectroscopy to determine the molecular weight of PLA samples and compare them to those obtained from viscometry. 

NMR experiments were recorded at 298 K on a Bruker Avance NEO NMR spectrometer (Bruker, Billerica, MA, USA) equipped with a 9.4 T magnet, GAB/2 gradient amplifier and 5 mm Prodigy cryoprobe with z-gradient coil with maximum gradient strength of 50 G cm^−1^, operating at 400.20 MHz (^1^H). Deuterochloroform was used as solvent and PLA concentrations were in the range of 0.5–1.0 mg/mL to neglect the impact of polymer chain interactions on the diffusion coefficients. Chloroform is a low-viscosity solvent and is susceptible to convection currents; thus, all experiments were run without spinning to reduce convection. In addition, a pulse sequence that minimizes magnetization decay due to translational motion arising from convection currents associated with temperature gradients across the sample was used. The BRUKER *dstebpg3s* pulse sequence incorporates a double stimulated echo (dste) segment with three spoil gradients to suppress convection currents [69].

### 2.7. Mechanical Characterization Films

**Figure 3:**
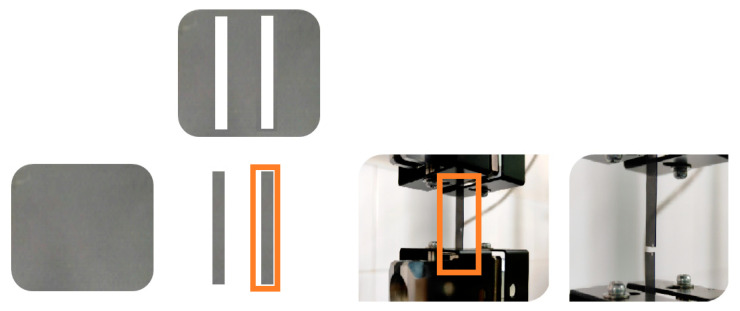
Set-up for tensile testing of film specimens.

The mechanical properties were evaluated by means of tensile test measurements using a Shimadzu AGS-X 100 N universal tensile testing machine (Shimadzu Corporation, Kyoto, Japan) equipped with a 100 N load cell, with an initial length of 30 mm and a crosshead speed of 5 mm/min. Films were cut into rectangular specimens 60 mm long and 5 mm wide using a razor guided by a straight edge. The thickness of each specimen was determined from the average of five measurements using a film thickness gauge. Sandpaper was fixed using adhesive to the ends of each specimen to prevent slipping while in the clamps of the materials testing machine (Figure 3). Between seven and nine specimens were used for each formulation. Young modulus, the ultimate tensile strength, and the elongation at break were calculated from the obtained stress–strain curves, and the media of at least five specimens was reported.

### 2.8. Mechanical Recycling Models

To analyze if the quality of the resulting material from mechanical recycling has a representative impact on the number of cycles that it can reach and if it is of importance to properly trace the origins of the material, the authors propose a model which allows one to review the different collection and mechanical recycling cycles under two different scenarios: (i) a full traceability of the PLA 3D printing waste from a single known source and, (ii) when considering PLA 3D printing waste from unknown sources. In this model, the discarded PLA, usually considered as waste, becomes an input for the next cycle and the system could regard it as a remanufactured product.

The characterization of the recycled and remanufactured product follows the case presented by Van Loon et al. [70], in which they describe the difficulties for collection and assume that recovering products would be a tedious task with high economic implications. Moreover, some of these products might end-up in the second-hand market due to their economic value. Contrarily, in the case proposed in this research, we considered that the second-hand market became irrelevant as the economic value of a discarded 3D printed product is equivalent to waste, given the particularities of the material and the high customization of 3D printed products; therefore, we weighed it directly as waste. It is worth noting that only the post-consumer (PC) waste part of the solid polymer waste (SPW) [24] was examined. PLA waste was considered to be any product that has not been properly printed (due to any kind of failure) and needed to be discarded, as well as all 3D printed support structures which keep the product stable and in-place during the printing process but are not part of the final product. Therefore, for our assumption, the printed product (D) and the waste (w) could be unified under the hypothesis that these can be joint together ($D^{\prime }$) as presented in Equation (6):

(6) $$D+w=D^{\prime }$$

Then, we ensure that:

(7) $$\left(\frac{D^{\prime }}{D}\to 1\right)\;when\;\frac{w}{D}\to 0$$

Additionally, we made two important considerations:

- the collection rate $\left(c\right)$ was not maximized $\left(c<1\right)$, meaning that some material could be lost or not fully collected and,
- the recycling rate $\left(r\right)$ was close to optimal rate, suffering just minor losses during the process $\left(r\;\sim \;1\right)$.

*Case 1: Full Traceability*

**Figure 4:**
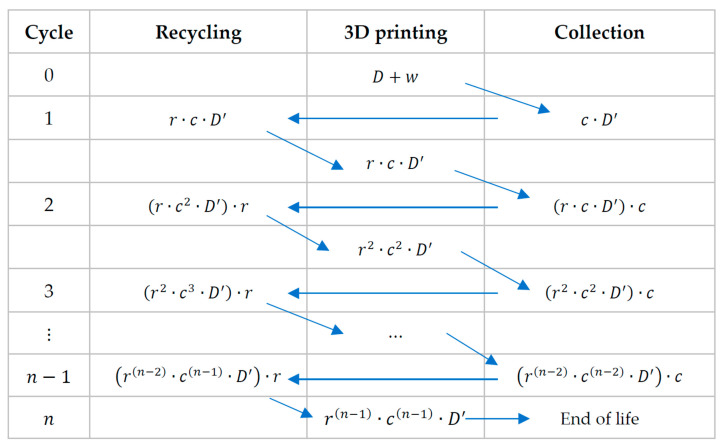
Scheme of mechanical recycling for full traceability (adapted from [70]).

For the first case, we proposed the review of a full traceability model under which the PLA is fully traced since its purchase as 3D printing filament (considered as “virgin” raw material) [57], and through all different processes, such as its use on product printing (D) and the collection of all residues (w) generated during the process. Then, the material is recovered, and the process of mechanical recycling begins. This process is represented in the following scheme (Figure 4) where the different mechanical recycling cycles and collection cycles are presented. 

The $n^{th}$ cycle is considered as the cycle on which the quality $\left(q\right)$ of $D^{\prime }$ decays considerably and will not be able to advance one further mechanical recycling cycle, therefore, we can declare the End of Life (EoL) for the material.

*Case 2: Not Full Traceability*

Comparatively, we presented a second case in which the materials came from several unknown sources, and it was impossible to properly differentiate “virgin” materials (without any mechanical recycling cycles) from recycled materials (independent of the number of cycles). For this, we considered the different sources and the different recycled cycles independent of each other. 

Equation (8) is presented as an update of Equation (6) in which the different sources (i) are identified and included, although these might be still considered as a first printing:

(8) $${\left(D+w\right)}_{i}=D_{i}^{\prime }$$

Additionally, a new variable (P) must be included to identify products (and its waste) that have undergone, at least, one recycled cycle, as presented in Equation (9), and are later included in the mechanical recycling process.

(9) $$P+w_{p}=P^{\prime }$$

**Figure 5:**
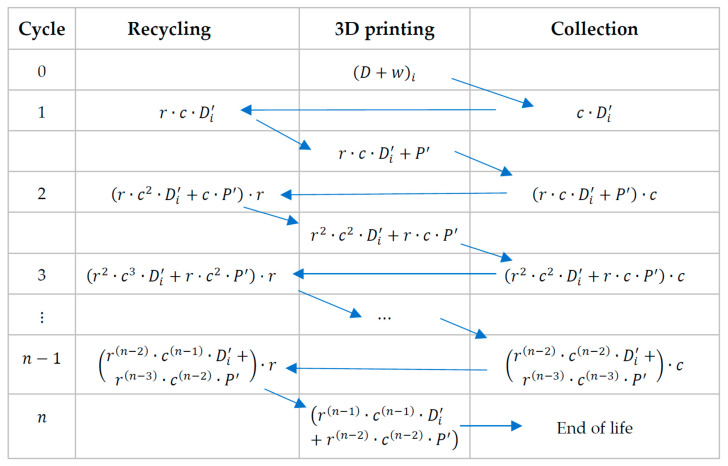
Scheme of mechanical recycling for not full traceability (adapted from [70]).

Maintaining the remainder of the mechanical recycling process as in the previous scheme (Figure 4), but introducing these new equations into it, we proposed the following scheme, shown in Figure 5:

The $n^{th}$ cycle is considered as the cycle in which the collective quality (q) of $\left(P^{\prime }+D_{i}^{\prime }\right)$ decays considerably and will not be able to advance one further mechanical recycling cycle, therefore, we can declare the End of Life (EoL) for the material.

*The Algorithm Behind the Mechanical Recycling Models*

Although the models seem complex mathematically, a simplistic approach to understand the theoretical models for each case is proposed:

*Case 1: Full Traceability*

The following recurrent algorithm consists of few steps to ensure that the virgin material is properly traced and the number of possible recycling cycles is maximized:

1. Keep track of 3D printed products and waste ($D^{\prime }$).
2. Collect and sort $D^{\prime }$ per recycling cycle.
3. Perform a mechanical recycling cycle.
4. Review material printing quality (q) after completing step 3.
5. Repeat steps 1 to 4 until quality (q) decays and does not allow the material to continue to the next recycling cycle.
6. Declare EoL.

As it can be seen, the process is straightforward and assumes that each cycle is carried out independently from the other and, because of traceability, there will not be mixing of materials, maximizing the possibility of reaching a higher number of recycling cycles.

*Case 2: Not Full Traceability*

Similarly, we present a recurrent algorithm for the special case under which the material’s origins are not traced, and the number of recycled cycles experienced by the material are also unknown. The algorithm pretends to maximize the number of possible recycling cycles:

1. Collect random products and waste ($D_{i}^{\prime }$).
2. Asses the quality (q) of the material through hand inspection and sort accordingly to the best of one’s intuition.
3. If the quality (q) is still high, use it as material for the recycling cycle; otherwise, declare EoL for that material.
4. Perform mechanical recycling cycle.
5. Track recycled filament (if of interest).
6. Repeat steps 1 to 5 until quality (q) decays and does not allow the material to continue to the next recycling cycle.
7. Declare EoL.

Comparatively with the previous algorithm, is possible to observe that the process includes an additional step of an early classification to optimize the final product. However, this process assumes that different mechanical recycling cycles are being carried with different materials, sorting them out by quality.

## 3. Results

### 3.1. FTIR Characterization of the Samples

Fourier transform infrared spectroscopy (FTIR) was conducted on the samples to study their chemical structure and to evaluate structural changes caused by reprocessing. The characteristic bands of PLA [71,72] were shown in all the spectra: the strong band at 1757 cm^−1^ corresponding to the C=O bond stretching; the bands at 1187 cm^−1^ and 1092 cm^−1^ attributed to the asymmetric vibration of the ester group (O−C−O); and the bands at 2997 cm^−1^ and 2945 cm^−1^ assigned to the C−H stretching of $-CH_{3}$, among others. To obtain comparable results without the experimental influence, transmittance values of these characteristic peaks were normalized using the absorbance value at 1454 cm^−1^ [58]. A comparison of the PLA-C spectra after each extrusion cycle revealed similar patterns: 1-PLA-C and 3-PLA-C spectra were quite similar (Figure S1) and slight changes were observed after four and five extrusion cycles (Figure S2). Although some small variations in intensity could be observed, neither disappearance nor appearance of new bands were detected; more specifically, the band corresponding to the anhydride function (1845 cm^−1^) was not observed, and the carbonyl stretching band (1757 cm^−1^) was not shifted. Therefore, we excluded that a significant change in molecular structure took place upon reprocessing. Analysis of the IR spectra performed on the PLA-M samples, from the mixture of the 3D printing parts from the COVID-19 personal protective equipment, gave essentially the same results (Figure S3): similar transmittance patterns were obtained for all the samples, from 1-PLA-M to 4-PLA-M, showing all the characteristics bands for PLA.

Furthermore, the FTIR spectra of both series (PLA-C and PLA-M) were compared, trying to detect the presence of other polymeric materials in the PLA-M samples. However, FTIR characterization showed the absence of significant contamination from other plastics (Figure S4). 

### 3.2. Effect of Mechanical Recycling on the Intrinsic Viscosity and Molecular Weight of the Samples

**Figure 6:**
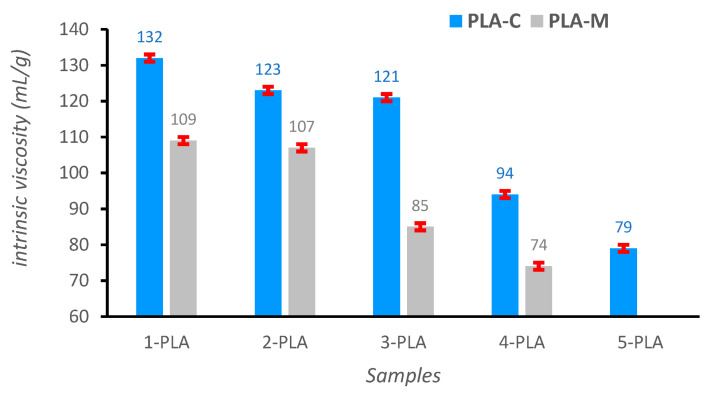
Intrinsic viscosity ($\left[\eta \right])$ values for the PLA samples ($CH{Cl}_{3},\;25\;℃).$

In 3D printing, viscosity is a very important parameter and must be in a certain range, low enough to allow extrusion at the operating temperature and high enough to provide structural stability [73]. The reduction in the intrinsic viscosity $\left(\left[\eta \right]\right)$ has already been reported for PLA samples subjected to mechanical recycling processes [27,55,74,75]. One of the objectives in this work was to compare PLA 3D printing wastes coming from a known source (PLA-C) with those from unknown sources (PLA-M). As it is shown in Figure 6, the intrinsic viscosity values decreased with increasing number of extrusion cycles, but at different rates. Thus, the intrinsic viscosity values for PLA-C, after three extrusion cycles, showed a reduction of only 8%, from 132±2 mL/g (1-PLA-C) to 121±2 mL/g (3-PLA-C) while for PLA-M, this decrease was more pronounced, 22%: from 109±1 mL/g (1-PLA-M) to 85±1 mL/g (3-PLA-M). Furthermore, the waste from the well-known reference grade presented, even after three extrusion cycles, higher values for intrinsic viscosity than 1-PLA-M: 121 mL/g (3-PLA-C) versus 109 mL/g (1-PLA-M). These results underline the importance of the sorting and separation stages in mechanical recycling, since the more inhomogeneous the sample, the worse the quality. 

It was also observed, in the case of the PLA of known origin, that while the viscosity values decreased slightly in the first three extrusion cycles (around 8%), there was a very sharp decrease after the fourth extrusion cycle (22%), from 121±2 mL/g (3-PLA-C) to 94±1 mL/g (4-PLA-C). A similar trend was noticed for the waste from unknown sources: slight decrease after the first two extrusion cycles (around 2%) and a pronounced decrease after the third extrusion cycle (21%). 

Our results from viscometry, seem to evidence the feasibility of using recycled PLA for additive manufacturing. However, due to the reduction in viscosity as a consequence of the material degradation, the number of reprocessing cycles seems to be limited to three for PLA-C and two for PLA-M.

**Figure 7:**
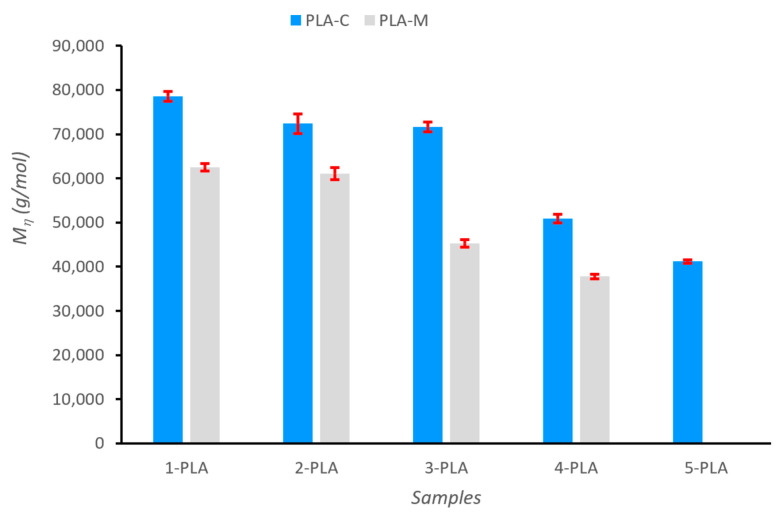
Viscosity-average molecular weight $\left(\bar{M_{\eta }}\right)$ for the PLA samples ($CH{Cl}_{3},\;25\;℃).$

The intrinsic viscosity $\left(\eta \right)$ is related to the viscosity-average molecular weight $\left(\bar{M_{\eta }}\right)$ of PLA (Figure 7) by the semi-empirical Mark–Houwink–Kuhn–Sakurada (MHKS) equation ([60,61,62]):

(1) $$\left[\eta \right]=2.21\times 10^{-4}\times {\bar{M_{\eta }}}^{0.77}\;\left(dL/g\right)$$

Since the first extrusion cycle, the samples from the mixture of PPE parts (PLA-M) showed lower molecular weights, around 20%, than the samples from the known origin (PLA-C): $7.86\cdot 10^{4}\;\pm 1\cdot 10^{3}g/mol$ for 1-PLA-C and $6.25\cdot 10^{4}\;\pm 8\cdot 10^{2\;}g/mol$ for 1-PLA-M. These differences can be attributed to the presence of different PLA grades in the 3D printing waste from the coronamakers, something completely logical as the waste, by its nature, is a mixture of PLA of different origins that have undergone different printing processes. 

The results indicated a strong reduction of molecular weight for PLA-C, up to 48% at the fifth reprocessing cycle; however, during the first three extrusion cycles, the reduction in molecular mass was only around 8.9% ($M_{1PLAC}=7.86\cdot 10^{4}\;\pm 1\cdot 10^{3}\;g/mol;$ $M_{3PLAC}=7.16\cdot 10^{4}\;\pm 1\cdot 10^{3}\;g/mol).$ Similarly, molecular weight for PLA-M reduced around 39.5% at the fourth extrusion cycle, but only 2.4% during the first two extrusion cycles ($M_{1PLAM}=6.25\cdot 10^{4}\;\pm 8\cdot 10^{2}\;g/mol;$ $M_{2PLAM}=6.10\cdot 10^{4}\;\pm 1\cdot 10^{3}\;g/mol).$ Botta et al. [76] reported a similar trend, to the one we observed, for the variation of molecular weight as a function of the number of reprocessing cycles.

We conclude that recycling into filaments for 3D printers seems feasible up to three extrusion cycles for PLA-C and two for PLA-M. Drastic reductions in molecular weights for PLA-C after the fourth cycle (35%) and for PLA-M after the third cycle (28%), did not allow further cycles of extrusion. Thus, recycled PLA with average viscous molecular weight $\bar{M_{\eta }}\geq 6\times 10^{4}\;g/mol$ are suitable for use in 3D printing.

### 3.3. Characterization by Nuclear Magnetic Resonance (NMR): Diffusion-Ordered (DOSY) Spectroscopy

**Figure 8:**
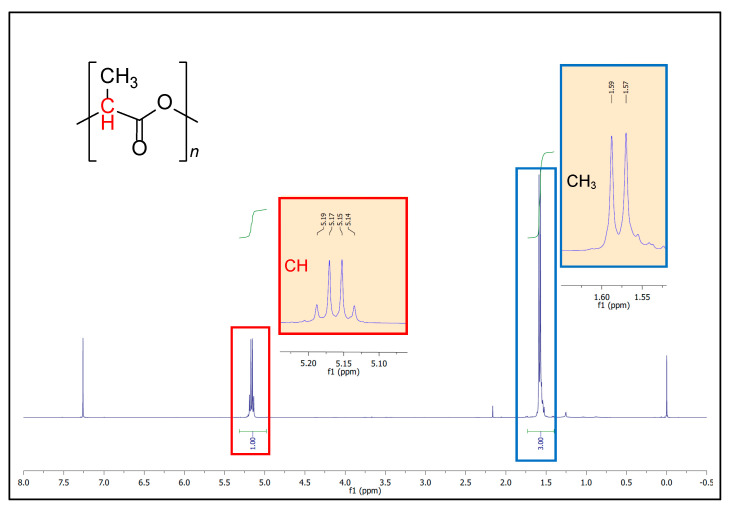
^1^H NMR spectrum for poly(lactic acid) in CDCl_3_ and expansion of the regions showing the CH and CH_3_ protons. The chemical shifts of TMS (0 ppm) and residual solvent signals (7.26 ppm) are also visible.

The ^1^H NMR spectrum for poly(lactic acid) in CDCl_3_ is shown in Figure 8:

The signals for the methyl group (CH_3_) and for the tertiary CH can be easily identified: a signal at 5.16 ppm corresponding to the tertiary CH as a quartet (q, *J* = 8 Hz, 1H) and a signal at 1.58 ppm for the CH_3_ group as a doublet (d, *J* = 8 Hz, 3H). The chemical shifts were referenced to the internal residual solvent signal at 7.26 ppm.

**Figure 9:**
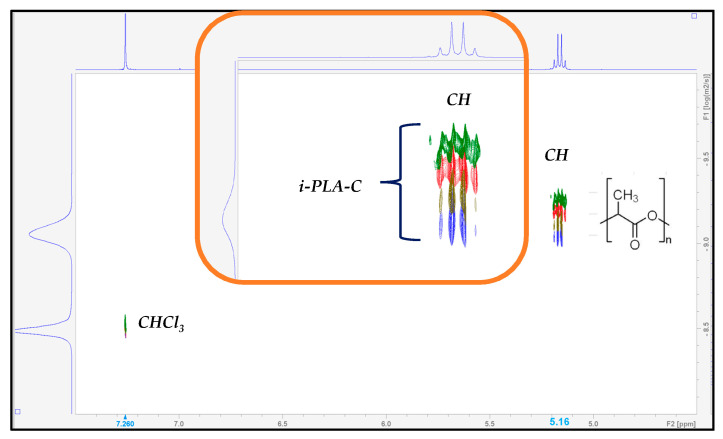
400 MHz 2D DOSY NMR stacked spectra obtained at 298 K in CDCl_3_ for the 3D printing waste PLA-C.

Being the determination of molecular weights of supreme relevance in the analysis of polymers, in this study we have tested the diffusion-ordered spectroscopy (DOSY) method for our PLA samples. Even though size exclusion chromatography (SEC) is, by far, the most used technique to characterize polymer molecular weight and molecular weight distribution, it still has some limitations [65]. In addition, some recent studies [77] have highlighted the potential use of DOSY for molecular weight analysis of a variety of consumer plastic products. DOSY is an NMR method that reports diffusion coefficients (D) for individual resonances in the NMR spectra. Due to the linear correlation (Equation (5)) of the logarithm of the diffusion coefficient (logD) to the logarithm of the molecular weights (logM), DOSY allows for molecular weight determinations. In a bidimensional (2D) DOSY spectra, as the one shown in Figure 9, the horizontal axis corresponds to the ^1^H NMR spectrum (F2, ppm), while the vertical axis corresponds to the logarithm of the translational diffusion coefficients (F1, $m^{2}/s).$

In our case, as mechanical degradation of the PLA-C samples reduces the average molecular weight of the polymer, different diffusion coefficients should be expected for the different samples. The higher the molecular weight, the lower the diffusion rate.

The DOSY map (Figure 9) shows an expanded view of the DOSY experiment (4.5–7.5 ppm region) for the samples of PLA-C. The signals corresponding to the solvent (7.26 ppm) and to the tertiary CH of PLA at 5.16 ppm are shown (F2). The largest diffusion coefficient is for the small molecule of solvent (CHCl_3_), at the bottom of the chart ($D_{CH{Cl}_{3}}=2.95\cdot 10^{-9}\;m^{2}/s;logD=-8.53)$. A superposition plot of four (out of the five) DOSY spectra of *i*-PLA-C is depicted in different colors, to better illustrate the distinct diffusion coefficients for the PLA samples. The remaining spectrum was not included in the stacked plot, for clarity. DOSY spectra were processed by Topspin 4.2.0 software and the diffusion coefficients are summarized in Table 2.

In this study, diffusion coefficients for the PLA-C samples have been obtained, showing the expected trend, larger molecules diffuse more slowly. Thus, diffusion coefficients range from the lowest value $\left(D_{1PLAC}=5.62\cdot 10^{-10}\;m^{2}/s;\;logD_{1PLAC}=-9.25\right)$ for the largest molecule $\left(M_{1PLAC}=7.86\cdot 10^{4}\;g/mol\right)$ to $D_{5PLAC}=8.32\cdot 10^{-10}\;m^{2}/s\;(logD_{5PLAC}=-9.08)$ for the PLA-C with the lowest molecular weight, 5-PLA-C ($M_{5PLAC}=4.12\cdot 10^{4}\;g/mol)$. There is a variation of around 48% for both diffusion coefficients and molecular weights.

**Figure 10:**
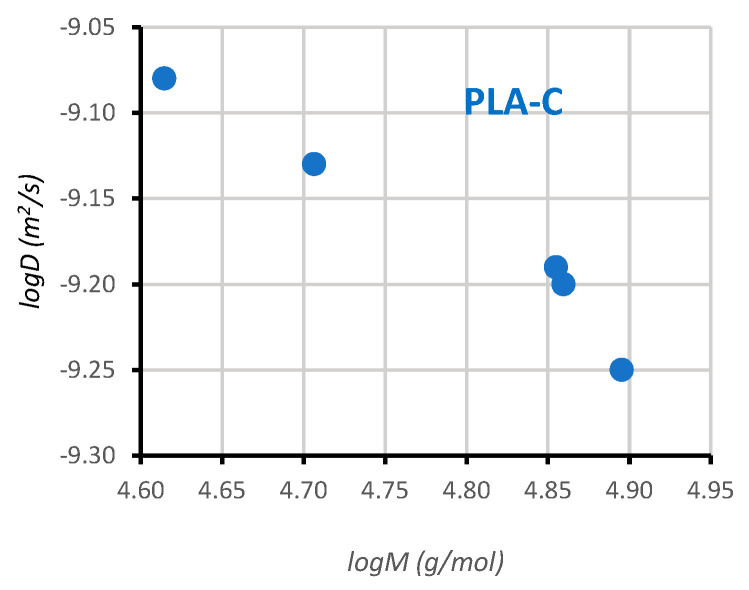
Plot of logD versus logM for PLA-C samples.

When the logarithm of the obtained diffusion coefficient was plotted against molecular weight (Figure 10), the following linear relationship was obtained (Equation (10)):

(10) $$logD=-0.5349\times logM-6.61\;(r^{2}=0.9515,\;D\;in\;m^{2}/s,\;M\;in\;g/mol)\;$$

Although the relationship does not seem to be perfectly linear, it should be noted at this point that the PLA samples used in this study are very different from the commercial standards used for calibrations. Nevertheless, the data from molecular weight and diffusion coefficients for the PLA-C samples correlate reasonably well. Thus, being aware of the many limitations of our study, we would like to highlight the potential use of DOSY for molecular weight analysis, provided the correct pulse sequence to reduce solvent convection effects is used and sufficiently diluted solutions of polymers are prepared.

### 3.4. Mechanical Characterization of the Film Tensile Tests

The viscosity and diffusion measurements have shown that repeated cycles of extrusion provoke a reduction in molecular weight. PLA films were prepared by compression molding to evaluate the mechanical properties and tensile tests were performed. Their mechanical performance was analyzed in terms of Young modulus (E), tensile strength (TS), and elongation at break ($\varepsilon_{b})$. Table 3 shows these results.

**Figure 11:**
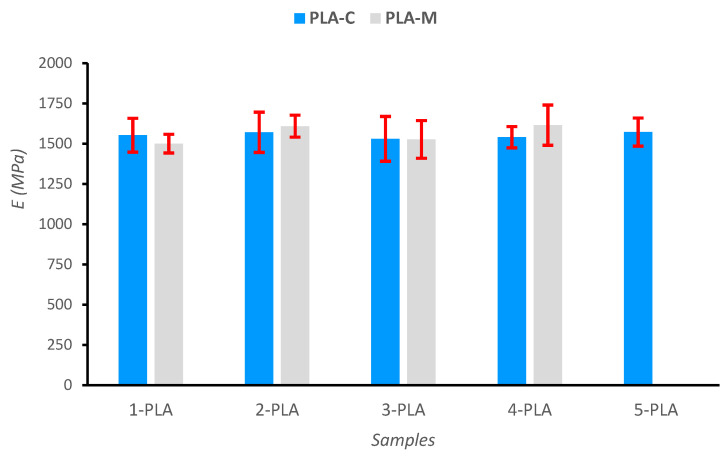
Tensile test: Young´s modulus for the different PLA films.

As can be seen from Table 3, there were no major differences in the elastic modulus for both types of printing wastes, PLA-C and PLA-M, with values in the range of 1500–1615 MPa. Similarly, Young’s modulus (E) did not noticeably vary with the number of extrusion cycles; thus, in the i-PLA-C series (films from the 3D printing waste of known origin) the change in the elastic modulus after the five extrusion cycles was around 1%: from 1553±105 MPa(1-PLA-C) to 1572±87 MPa (5-PLA-C) or $E_{5PLAC}/E_{5PLAC}=1.01.$ In addition, the maximum difference among the values was about 2.7% (without considering standard deviations). In the case of the films coming from the blend of printing wastes (i-PLA-M series), the change in Young’s modulus was somewhat higher, around 7.6%, from 1500±58 MPa(1-PLA-M) to 1615±125 MPa (4-PLA-M), which corresponds to the maximum difference values. Nevertheless, as the standard deviation is high, all results fall into the same value range, and no trends can be asserted (Figure 11). Although the main consequence of the recycling process is the reduction of molecular weight of PLA due to chain scission, the effect of the repeated extrusion cycles on the Young’s modulus for the films is negligible, as pointed out by other authors [48,49,75,78,79]. These results might be explained by the competition between two opposing phenomena during reprocessing: decrease of molecular weight and increase of crystallinity [75].

Considering the tensile strength (TS)**,** PLA-C showed higher values than PLA-M ($\sigma_{1PLAC}=46.3\pm 5.9\;MPa$ versus $\sigma_{1PLAM}=33.4\pm 4.5\;MPa).$ A reduction in each recycling process was observed for the PLA-C series (Table 3), representing in total a percentage reduction of around 26% at the fifth reprocessing cycle (without considering standard deviations); on the other hand, tensile strength for PLA-M, kept constant until the third extrusion cycle. The elongation at break (ε) progressively decreased down from initial values ($\varepsilon_{1PLAC}=4.08\pm 0.60$ and $\varepsilon_{1PLAM}=3.50\pm 0.90)$ to ($\varepsilon_{5PLAC}=3.31\pm 1.18$ and $\varepsilon_{4PLAM}=2.06\pm 0.20)$. In any case, these low values are characteristic for brittle materials [80]. 

The decrease of tensile strength might be attributed to a lower cohesion in the materials, according to Pillin et al. [81], while the progressive diminution of elongation at break was explained by the simultaneous decrease of the molecular weight and the increase of crystallinity, favouring crack propagation.

## 4. Conclusions

This work describes the effect of multiple reprocessing cycles on the properties of PLA 3D printing wastes from different origins. The wastes evaluated here came from a single, well-known commercially available source (PLA-C) and from a mixture (PLA-M) of parts (masks, visors, other components) of the personal protective equipment generated by an association of coronamakers in Madrid, during COVID-19. FTIR analysis of both series (PLA-C and PLA-M) showed the characteristic pattern of PLA, thus excluding significant contamination from other plastics. After an initial washing step, both types of waste were shredded, dried, and extruded into filaments, part of the extruded filaments was separated for characterization and preparation of films by compression molding, and the remaining material was subjected to repeated extrusion cycles. Reprocessing was continued until degradation of the material did not allow further cycles of extrusion, which occurred for PLA-C at the fifth cycle and for PLA-M at the fourth cycle. 

Intrinsic viscosity measurements and molecular weight analysis indicated a strong reduction of molecular weight for both 48% at the fifth reprocessing cycle for PLA-C and 39.5% at the fourth extrusion cycle for PLA-M. However, more interestingly, during the first three extrusion cycles, reduction in molecular mass was only around 8.9% for PLA-C; and in the case of PLA-M, only 2.4% for the first two extrusion cycles. However, pronounced decreases for both molecular weights were observed after the fourth cycle (35% reduction, PLA-C) and the third cycle (28% reduction, PLA-M), respectively. Therefore, we conclude that recycling wastes into filaments for 3D printers seems feasible up to three extrusion cycles for PLA-C and two for PLA-M, provided average viscous molecular weight $\bar{M_{\eta }}\geq 6\times 10^{4}\;g/mol$. 

Diffusion-ordered NMR spectroscopy (DOSY) was used to estimate molecular weights; diffusion coefficients were obtained using the BRUKER *dstebpg3s* pulse sequence to suppress convection currents of solvent and by working at high dilution conditions (0.5−1.0 mg/mL). In these conditions, a linear correlation between diffusion coefficients and molecular weights was observed, which confirms the potential of the technique.

Mechanical properties of the PLA films were in concordance with published results, as no variation of elastic modulus was observed, but decrease of tensile strength and elongation at break occurred during reprocessing. PLA-C films showed a slightly better performance than PLA-M films.

Overall, our results indicate that recycling 3D printing waste into filaments is feasible, to some extent, and we especially recommended this technique in university communities, which, like ours (Polytechnic University of Madrid, Universidad Politécnica de Madrid, UPM) are actively involved in circular economy projects.

There remains one point that needs further investigation, whether it is preferable to limit recycling to 3D printing waste from a single source or to use PLA wastes from different sources. The fact that in our study the difference is of only a cycle, raises doubts about which is the best option, since PLA-M offers advantages of economic type, a higher quantity of material and easy material collection. The mechanical recycling model proposed here (full traceability/not full traceability) addresses this issue.

## Figures and Tables

**Table 1:** Materials used in this study.

Sample Code	Description of the 3D Printing Waste
**1-PLA-C**	**1** additional extrusion cycle- PLA **waste from commercial** filament
**2-PLA-C**	**2** additional extrusion cycles- PLA **waste from commercial** filament
**3-PLA-C**	**3** additional extrusion cycles- PLA **waste from commercial** filament
**4-PLA-C**	**4** additional extrusion cycles- PLA **waste from commercial** filament
**5-PLA-C**	**5** additional extrusion cycles- PLA **waste from commercial** filament
**1-PLA-M**	**1** additional extrusion cycle- PLA **waste from mixture** of PPE parts
**2-PLA-M**	**2** additional extrusion cycles- PLA **waste from mixture** of PPE parts
**3-PLA-M**	**3** additional extrusion cycles- PLA **waste from mixture** of PPE parts
**4-PLA-M**	**4** additional extrusion cycles- PLA **waste from mixture** of PPE parts

**Table 2:** Diffusion coefficients determined via DOSY for PLA-C samples from the ^1^H NMR signal at 5.16 ppm (CH chemical shift).

Sample Label	logM (g/mol)	logD $\left(m^{2}/s\right)$
**1-PLA-C**	4.90	−9.25
**2-PLA-C**	4.86	−9.20
**3-PLA-C**	4.85	−9.19
**4-PLA-C**	4.71	−9.13
**5-PLA-C**	4.61	−9.08

**Table 3:** Mechanical properties of the films obtained by compression molding from the PLA wastes.

Sample Label	E(MPa)	TS(σ,MPa)	$\varepsilon_{b}(\%)$
**1-PLA-C**	1553 ± 105	46.3 ± 5.9	4.08 ± 0.60
**2-PLA-C**	1571 ± 125	43.0 ± 2.8	4.10 ± 0.10
**3-PLA-C**	1530 ± 140	37.3 ± 4.6	3.55 ± 0.50
**4-PLA-C**	1540 ± 66	36.7 ± 8.3	3.73 ± 1.10
**5-PLA-C**	1572 ± 87	34.2 ± 3.6	3.31 ± 1.18
**1-PLA-M**	1500 ± 58	33.4 ± 4.5	3.50 ± 0.90
**2-PLA-M**	1610 ± 70	33.3 ± 5.0	3.41 ± 0.97
**3-PLA-M**	1527 ± 120	33.7 ± 3.9	3.78 ± 0.82
**4-PLA-M**	1615 ± 125	27.7 ± 2.1	2.06 ± 0.20

## Data Availability

The data presented in this study are available on request from the corresponding author.

## Supplementary Materials

The following supporting information can be downloaded at: https://www.mdpi.com/article/10.3390/polym15173651/s1, Figure S1: FTIR normalized spectra of 1-PLA-C and 3-PLA-C; Figure S2: FTIR normalized spectra of PLA-C series. Expansion of the region 1425–2125 cm^−1^; Figure S3: FTIR normalized spectra of PLA-M series. Expansion of the region 1425–2125 cm^−1^; Figure S4: FTIR normalized spectra of 1-PLA-C and 1-PLA-M.
