# Supplementary material for: Recycled PLA for 3D Printing: A Comparison of Recycled PLA Filaments from Waste of Different Origins after Repeated Cycles of Extrusion
Source: Polymers (Basel). 2023 Sep 4;15(17):3651. doi: 10.3390/polym15173651 (PMC10490016; doi:10.3390/polym15173651)
Supplement: Supplementary file 1 [file polymers-15-03651-s001.zip › polymers-2160255-supplementary.pdf]

polymers

Supporting Information

Recycled PLA for 3D Printing: A Comparison of Recycled PLA Filaments from Waste of Different Origins after Repeated Cycles of Extrusion

David Hidalgo-Carvajal [1,*], Álvaro Hortal Muñoz [1,2], José J. Garrido-González [3], Ruth Carrasco-Gallego [1] and Victoria Alcázar Montero [1,4,*]

[1] Escuela Técnica Superior de Ingenieros Industriales, Universidad Politécnica de Madrid, 28006 Madrid, Spain; ruth.carrasco@upm.es (R.C.-G.)
[2] Dirección de Compras Industrial y Cliente, Repsol, 28006 Madrid, Spain
[3] Facultad de C. Químicas, Universidad de Salamanca, 37008 Salamanca, Spain
[4] Grupo de Investigación Polímeros, Caracterización y Aplicaciones (POLCA), 28006 Madrid, Spain
* Correspondence: david.hidalgo.carvajal@upm.es (D.H.-C.); mariavictoria.alcazar@upm.es (V.A.M.)

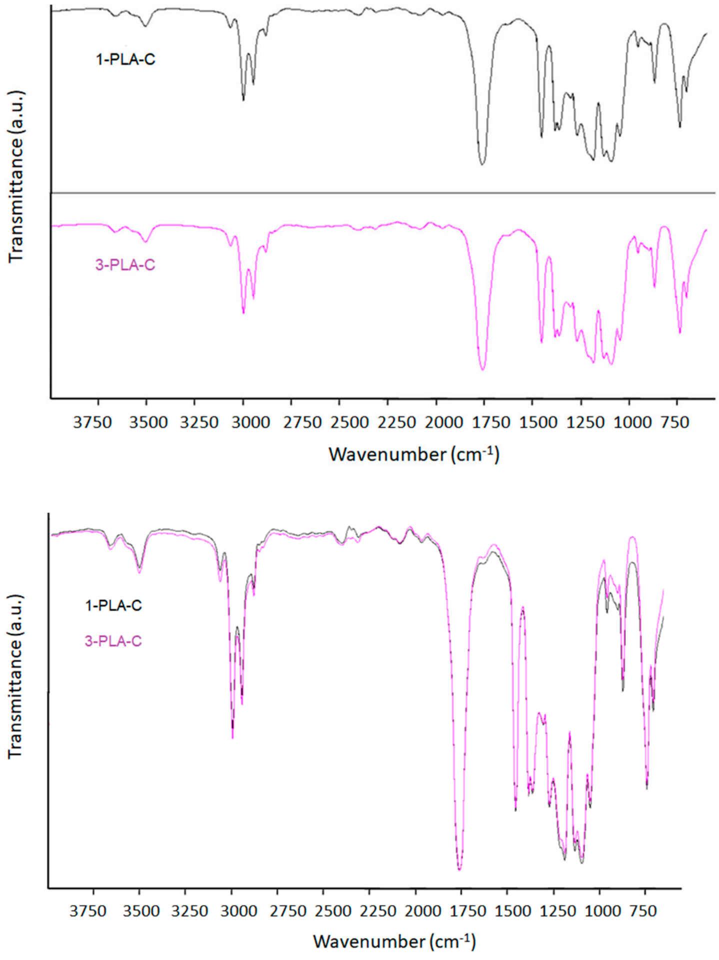

Figure S1. FTIR normalized spectra of 1-PLA-C and 3-PLA-C.

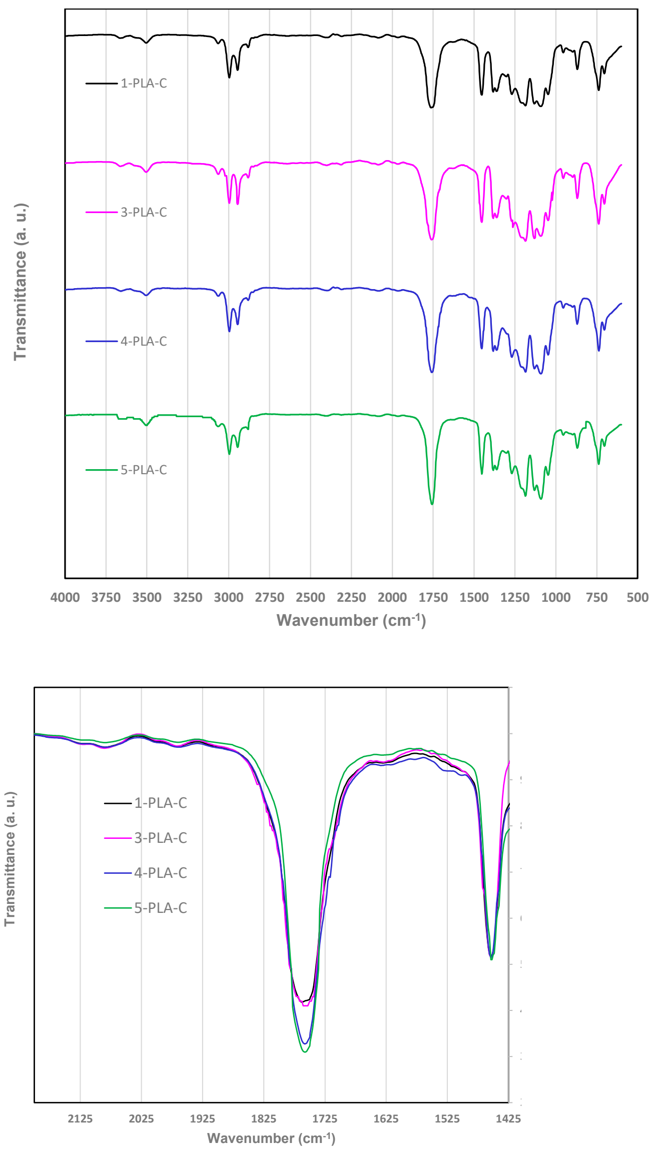

Figure S2. FTIR normalized spectra of PLA-C series. Expansion of the region 1425-2125 cm^{-1}.

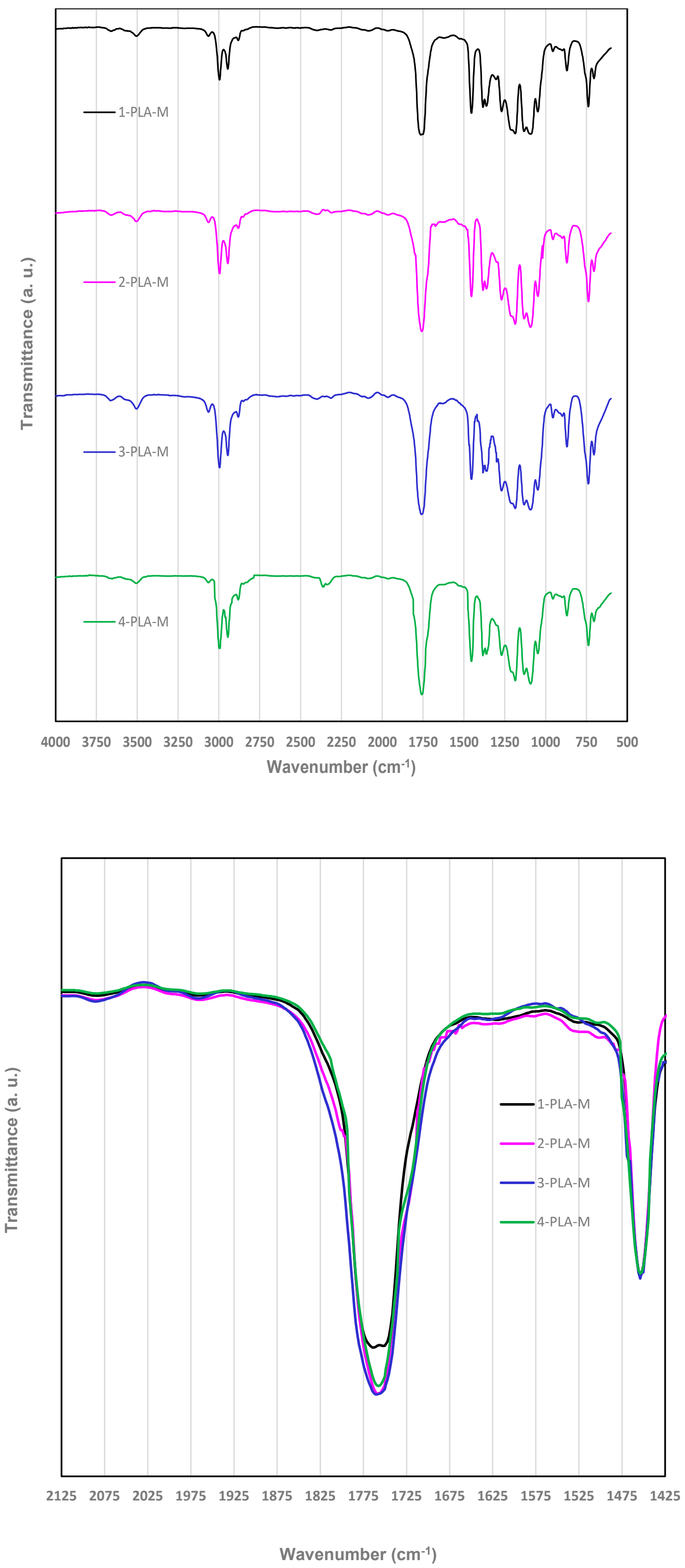

Figure S3. FTIR normalized spectra of PLA-M series. Expansion of the region 1425-2125 cm^{-1}.

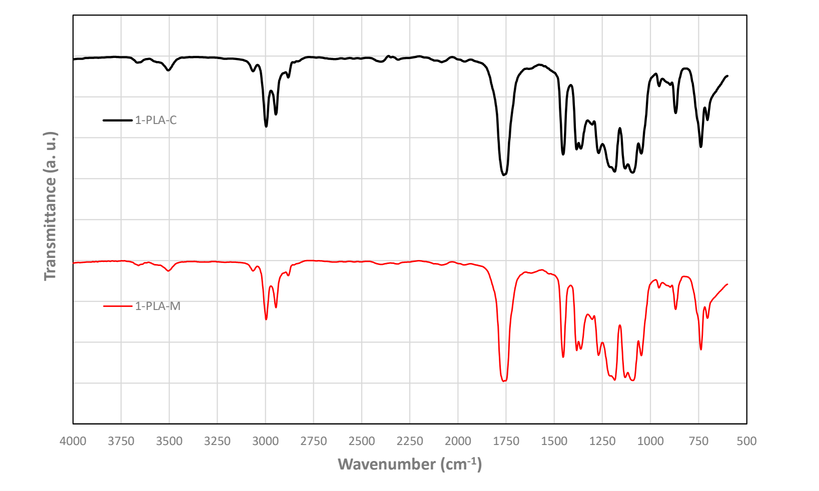

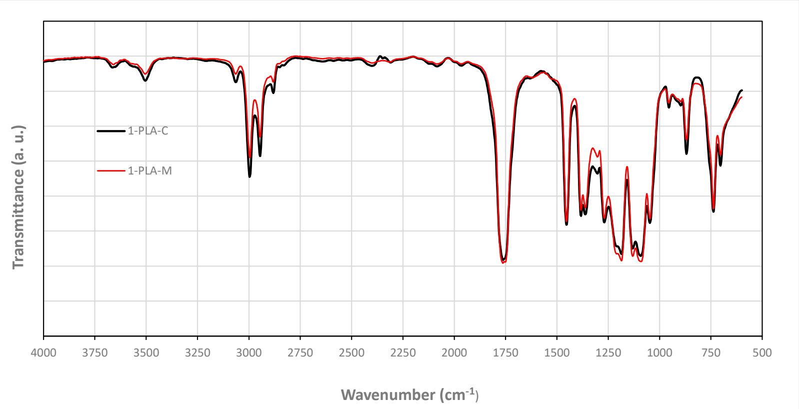

Figure S4. FTIR normalized spectra of 1-PLA-C and 1-PLA-M.
